# Statistically Optimized Tacrolimus and Thymoquinone Co-Loaded Nanostructured Lipid Carriers Gel for Improved Topical Treatment of Psoriasis

**DOI:** 10.3390/gels9070515

**Published:** 2023-06-25

**Authors:** Meraj Alam, Md. Rizwanullah, Showkat R. Mir, Saima Amin

**Affiliations:** 1 Department of Pharmaceutics, School of Pharmaceutical Education and Research, Jamia Hamdard, New Delhi 110062, India; merajalam1526@gmail.com (M.A.); mdrizwanullah54@gmail.com (M.R.); 2 Department of Pharmacognosy and Phytochemistry, School of Pharmaceutical Education and Research, Jamia Hamdard, New Delhi 110062, India; srmir@jamiahamdard.ac.in

**Keywords:** tacrolimus, thymoquinone, nanostructured lipid carrier, Box–Behnken design, psoriasis, topical delivery

## Abstract

The aim of this investigation was to develop and analyze a tacrolimus and thymoquinone co-loaded nanostructured lipid carriers (TAC-THQ-NLCs)-based nanogel as a new combinatorial approach for the treatment of psoriasis. The NLCs were formulated by an emulsification-solvent-evaporation technique using glyceryl monostearate, Capryol 90 (oil), and a mixture of Tween 80 and Span 20 as a solid lipid, liquid lipid, and surfactant, respectively. Their combination was optimized using a three-factor and three-level Box–Behnken design (3^3^-BBD). The optimized TAC-THQ-NLCs were observed to be smooth and spherical with a particle size of 144.95 ± 2.80 nm, a polydispersity index of 0.160 ± 0.021, a zeta potential of −29.47 ± 1.9 mV, and an entrapment efficiency of >70% for both drugs. DSC and PXRD studies demonstrated the amorphous state of TAC and THQ in the lipid matrix of the NLCs. An FTIR analysis demonstrated the excellent compatibility of the drugs with the excipients without interactions. The TAC-THQ-NLC-based nanogel (abbreviated as TAC-THQ-NG) exhibited a good texture profile and good spreadability. The in vitro release study demonstrated a sustained drug release for 24 h from the TAC-THQ-NG that followed the Korsmeyer–Peppas kinetic model with a Fickian diffusion mechanism. Moreover, the TAC-THQ-NG revealed significantly higher dose-dependent toxicity against an HaCaT cell line compared to a TAC-THQ suspension gel (abbreviated as TAC-THQ-SG). Furthermore, the developed formulations demonstrated antioxidant activity comparable to free THQ. Confocal microscopy revealed improved permeation depth of the dye-loaded nanogel in the skin compared to the suspension gel. Based on these findings, it was concluded that TAC-THQ-NG is a promising combinatorial treatment approach for psoriasis.

## 1. Introduction

Psoriasis is one of the most common autoimmune diseases characterized by chronic skin inflammation. It affects an average of 2 to 4% of the global population [1]. This awful condition is characterized by itchy, scaly, and flaky skin. It is also associated with swelling, discomfort, and disfiguring lesions on the skin [2]. Psoriasis pathogenesis is very complicated and dynamic. It may result from skin trauma, stress, infections, and some medications. It is also caused by different environmental factors, and some people may be genetically predisposed to it [3]. Approximately 80% of patients with psoriasis have mild to moderate symptoms that are typically managed with topical treatments. Those with severe disease are treated with phototherapy and systemic therapies [4]. Currently, topical therapy remains the preferred route of treatment in the management of psoriasis due to inherent advantages, such as better patient compliance, localized therapeutic action, and minimal systemic toxicity [5]. However, topical treatments also suffer from the major drawback of inadequate drug penetration through the scaly psoriatic skin. Thus, a carrier system that is able to deliver a therapeutic quantity of drug through the diseased tissues still remains a subject of research related to anti-psoriatic therapy [6].

Tacrolimus (TAC), a macrolide immunosuppressant derived from *Streptomyces tsukubensis*, has been widely used for the treatment of psoriasis-like skin disease. It is known to decrease the expression of pro-inflammatory cytokines and, thereby, inflammation [7]. However, its ineffectiveness as a topical treatment for psoriasis is attributed to its inability to penetrate the skin due to its high hydrophobicity and large molecular weight. Additionally, TAC-based ointments suffer from various drawbacks, such as variable absorption, skin irritations, redness, and pain, thus limiting their application as a topical anti-psoriatic agent [8]. Thymoquinone (THQ) is a lipid-soluble benzoquinone that is the principal bioactive constituent of black cumin (*Nigella sativa*) seeds. THQ has been found to exhibit outstanding antioxidant, anti-inflammatory, and anticancer effects in both in vitro and in vivo settings. It also exhibits free- and superoxide-radical scavenging action by potentiating the activity of several endogenous antioxidant enzymes [9]. Furthermore, its anti-psoriatic potential was evaluated in different studies with promising results [10,11]. Despite having therapeutic advantages, THQ′s pharmaceutical application is restricted because of its high hydrophobicity, poor solubility, low bioavailability, and inadequate penetration [12].

Several single-drug-based topical formulations are available on the market, but none are able to cure psoriasis effectively. Therefore, combinatorial therapy for better psoriasis management is often advised [13]. The idea behind using a drug combination is that the drugs may act through different pathways, thus providing a multi-targeted approach with improved efficacy. It is also associated with a reduction in dose-related adverse effects [14]. Due to these advantages over monotherapy, combination-based treatments have drawn the attention of research communities throughout the world. New combination formulations provide patients with the efficacy and safety advantages of two or more drugs simultaneously. Combinatorial formulations also exhibit greater efficacy and lower recurrence after the treatment is terminated [15,16]. Given these facts, the current work considers the use of TAC and THQ acting via separate pathways and complementing each other as effective anti-psoriatic agents.

In addition to the above-mentioned challenges with anti-psoriatic drugs, the presence of the physiological barrier in the form of the *Stratum corneum* (SC) make things worse. This topmost epidermal layer acts as a major impediment to drug administration through the topical route, resulting in limited drug transfer across the skin layers, and, ultimately, lower bioavailability [17]. The issue of drug penetration through the skin layers must be addressed to achieve the required drug concentration in the skin tissues. The application of novel approaches, such as nanotechnology-based drug delivery systems, could be a better option to ensure sufficient drug delivery into the inner layers of the skin. The use of lipid-based nanocarriers for the topical delivery of bioactive drugs has been widely reported. These carriers show excellent potential in managing different skin diseases due to their ability to interact with the skin at the molecular level and modify its barrier properties [18,19]. Among lipid-based nanocarriers, nanostructured lipid carriers (NLCs) are considered to be the most efficient nanocarrier for topical anti-psoriatic agents [20]. NLCs not only improve the dissolution rate of the encapsulated drug but are also easily permeated across the SC due to their small size, thus improving the overall bioavailability of lipophilic drugs [21]. NLCs also provides additional advantages, such as greater drug loading, improved release properties, and higher stability of incorporated drugs during storage, as compared to other lipid-based formulations [22]. Another major advantage of nanocarriers is their highly occlusive nature, which makes it possible for them to adhere completely to the skin and form a continuous film over the skin’s surface [21]. 

Therefore, our study focused on the fabrication of a TAC and THQ co-loaded NLCs (TAC-THQ-NLCs) and an evaluation of its potential efficacy against psoriasis. The developed formulation was optimized using a Box–Behnken design. A nanogel was formulated for ease of application. The developed formulation was characterized in terms of various parameters, such as in vitro drug release, cell culture, and a skin permeation study.

## 2. Results and Discussion

### 2.1. Estimation of TAC and THQ via RP-HPLC

**Figure 1:**
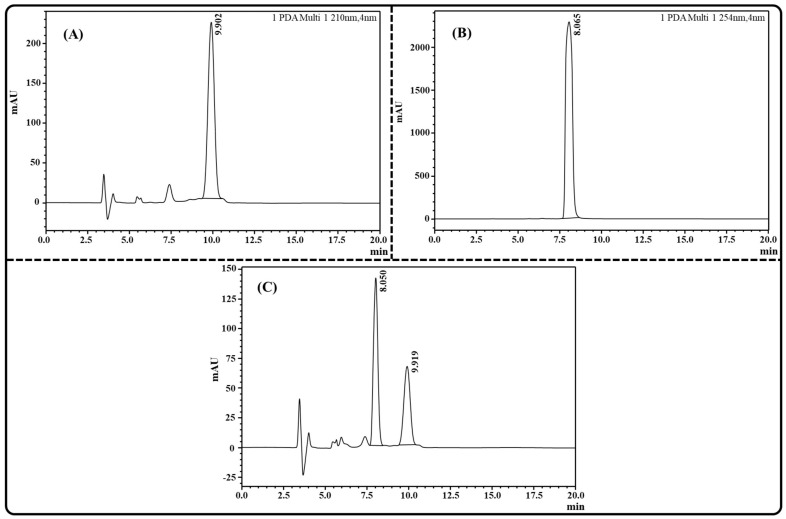
Image showing RP-HPLC chromatogram of: (**A**) TAC; (**B**) THQ; and (**C**) TAC and THQ for simultaneous estimation.

A robust, sensitive, and accurate RP-HPLC technique for simultaneous quantification of TAC and THQ was successfully developed and validated. The RP-HPLC chromatogram of TAC, THQ, and a mixture of TAC and THQ is depicted in Figure 1A–C. In this study, the linearity range of the developed RP-HPLC method for both TAC and THQ was found between 1 µg/mL and 80 µg/mL concentration range, and the correlation coefficient (R^2^) was observed to be 0.9989 and 0.9998 for TAC and THQ, respectively. The approximate retention time of the TAC and THQ was observed to be 9.9 and 8.0 min, respectively, at their respective wavelengths. The limit of detection (LOD) and limit of quantification (LOQ) for the quantification of the TAC was observed to be 0.065 µg/mL and 0.195 µg/mL, respectively, while for THQ, the LOD and LOQ were observed to be 0.021 µg/mL and 0.062 µg/mL, respectively. 

### 2.2. Excipients Screening

#### 2.2.1. Selection of Liquid Lipid (Oil), Solid Lipid, and Binary Mixture

**Figure 2:**
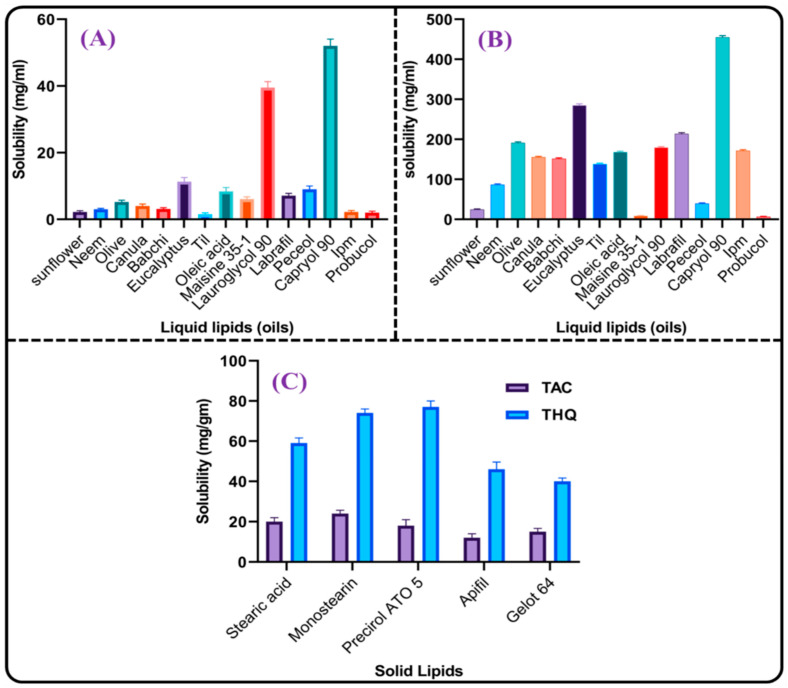
Solubility profiles of: (**A**) TAC in liquid lipids; (**B**) THQ in liquid lipids; and (**C**) TAC and THQ in solid lipids.

As drug solubility in lipids significantly influences the PS, %EE, and drug release of the formulation, it was important to determine drug saturation solubility in the lipids for the development of the NLCs [23]. The solubility profiles of TAC and THQ in different liquid lipids are depicted in Figure 2A,B. Both drugs (TAC and THQ) demonstrated maximum solubility in Capryol 90. TAC and THQ showed solubilities of 52 ± 2.09 mg/mL and 455 ± 4.31 mg/mL, respectively, in Capryol 90. Therefore, based on maximum solubility, Capryol 90 was chosen as a liquid lipid. The solubility profiles of TAC and THQ in various solid lipids are depicted in Figure 2C. TAC showed maximum solubility in GMS (24 ± 1.73 mg/gm), and THQ showed solubilities of 74 ± 2.00 and 77.33 ± 3.06 mg/gm in GMS and Precirol ATO 5, respectively. Thus, based on the solubility profiles of both drugs in lipids, Capryol 90 and GMS were chosen as liquid and solid lipids, respectively, for the preparation of the NLCs. Among the various ratios of the GMS and Capryol 90 evaluated for miscibility, as shown in Table 1, GMS showed good miscibility with Capryol 90 and showed no signs of phase separation at higher concentrations of oil after solidification. On the basis of a visual observation of the phase separation, a binary mixture (GMS and Capryol 90) in the ratio of 6:4 *w*/*w* was chosen for the fabrication of the TAC-THQ-NLCs. 

#### 2.2.2. Selection of Surfactant and Co-Surfactant

The stability of the emulsion and its capacity to emulsify were taken into consideration when choosing the surfactants. A higher %transmittance indicates smaller particles, improved physical stability, and non-aggregation of lipid nanoparticles, hence better emulsification. Table 2 shows the %transmittance (%T) of a lipid mixture created using different surfactants. It was observed that for the binary lipid mixture, Tween 80 showed the highest emulsification capacity; therefore, it was selected as a principal surfactant; however, previous research reports have shown that a combination of surfactants with one having a higher HLB and the other a lower HLB is always superior to a single surfactant in the production of smaller-sized nanoparticles with improved storage stability [24]. Therefore, Tween 80 and Span 20, having HLB values of 15 and 8.6, respectively, were chosen as a surfactant and co-surfactant due to their good emulsification capacity for the preparation of the TAC-THQ-NLCs. The non-ionic nature of surfactants also means that they are generally considered non-toxic for dermal application. Furthermore, its membrane-fluidizing property means that Tween 80 has the added benefit of enhancing the skin permeation of hydrophobic drugs [25]. 

### 2.3. Formulation and Optimization of TAC-THQ-Loaded NLCs by BBD 

**Figure 3:**
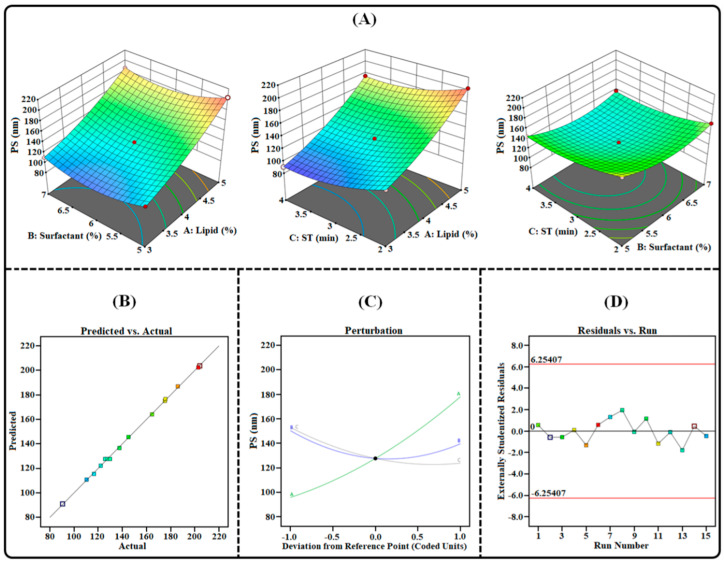
Image illustrating the impact of independent factors on PS: (**A**) 3D surface plot; (**B**) predicted vs. actual response; (**C**) perturbation plot; and (**D**) residual vs. run plot of TAC-THQ-NLCs optimized by BBD.

**Figure 4:**
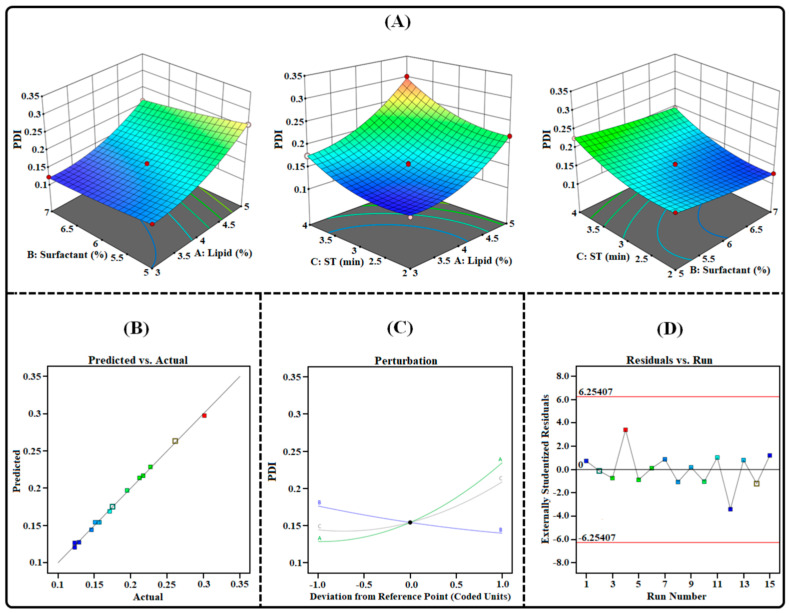
Image illustrating the impact of independent factors on PDI: (**A**) 3D Surface plot; (**B**) predicted vs. actual response; (**C**) perturbation plot; and (**D**) residual vs. run plot of TAC-THQ-NLCs optimized by BBD.

**Figure 5:**
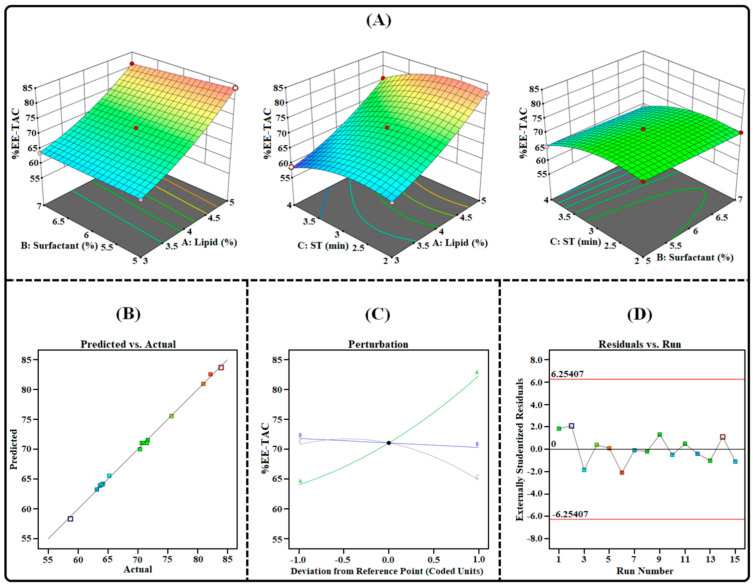
Image illustrating the impact of independent factors on the %EE-TAC: (**A**) 3D surface plot; (**B**) predicted vs. actual response; (**C**) perturbation plot; and (**D**) residual vs. run plot of TAC-THQ-NLCs optimized by BBD.

**Figure 6:**
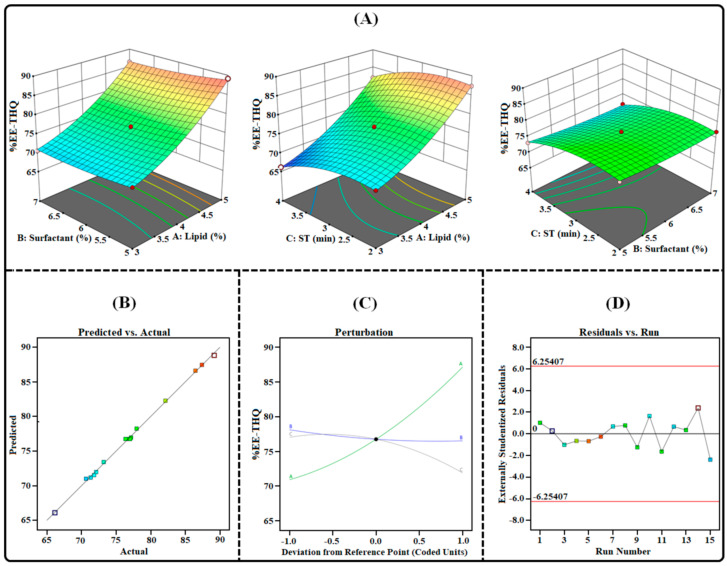
Image illustrating the impact of independent factors on the %EE-THQ: (**A**) three-dimensional surface plot; (**B**) predicted vs. actual response; (**C**) perturbation plot; and (**D**) residual vs. run plot of TAC-THQ-NLCs optimized by BBD.

The emulsification solvent-evaporation technique was successfully employed to prepare the TAC-THQ-NLCs. This technique of developing NLCs is simple and efficient and can produce particles in the nano-size range. In order to optimize the TAC-THQ-NLCs, 3^3^-BBD recommended 15 experimental compositions, as shown in Table 3. The influence of independent variables (*X*_1_, *X*_2_, and *X*_3_) on dependent factors (*Y*_1_, *Y*_2_, *Y*_3_, and *Y*_4_) was quantified and statistically evaluated by using analysis of variance (ANOVA). The regression analysis results for all four dependent factors were fitted independently into different models (linear, 2FI, cubic, and quadratic) to determine the best-fitting model having the highest adjusted and predicted R^2^, the results of which are summarized in Table 4. The findings showed that the suggested quadratic models demonstrated a strong fit to the data, with model terms being highly significant for all four dependent factors. These equations, which were based on data, used the quadratic polynomial model. All four dependent variables had “Predicted R^2^” values that were reasonably close to their corresponding “Adjusted R^2^” values. Three-dimensional surface plots, predicted vs. actual response plots, perturbation plots, and residual vs. run plots obtained for all the dependent factors are shown in Figure 3, Figure 4, Figure 5 and Figure 6. 

#### 2.3.1. Effect on Particle Size (*Y*_1_) 

The PS is one of the most essential quality parameters when it comes to the development of nanoformulations as it not only affects the rate of permeation but also determines how much of the drug is deposited in the skin. In order for a formulation to be effectively delivered to the skin, its particle size should be less than 200 nm [26]. Table 3 illustrates the particle-size distribution of the TAC-THQ-NLCs obtained from various experimental runs recommended by BBD. When different experimental runs were taken into account, it was discovered that the average particle size varied between 90.50 and 204.1 nm. The following is an expression for the computed polynomial equation for PS:

Particle Size (*Y*_1_) = +127.70 + 41.07 *X*_1_ − 5.36 *X*_2_ − 14.61 *X*_3_ − 3.02 *X*_1_*X*_2_ + 0.9750 *X*_1_*X*_3_ + 0.9000 *X*_2_*X*_3_ + 9.23 *X*_1_^2^ + 17.30 *X*_2_^2^ + 10.75 *X*_3_^2^ (1)

According to the coded quadratic Equation (1) and various plots as shown in Figure 3, the particle-size distribution of the TAC-THQ-NLCs changes when the levels of independent factors are altered. The overall effect of the concentration of the lipid mixture on the particle size is positive, which implies that when the percentage of lipid increases, there is a significant increase in the particle size of the NLCs. The reason behind such an increase in particle size is the lack of surfactant concentration, which may not be able to provide proper emulsification of the increased amount of lipid present in the aqueous phase of the formulation [27]. The overall effect of the surfactants was negative in terms of the particle size of the NLCs, due to the fact that when the concentration of the surfactant concentration is increased, the interfacial tension between the lipid and aqueous phase decreases, which leads to the formation of smaller-sized particles [28]. In addition, a higher concentration of surfactants up to a critical micelle concentration effectively stabilizes the NLCs by forming a steric barrier on their surface, protecting smaller NLCs and preventing their aggregation into larger ones [29]. As the sonication time increases, due to cavitation, high shear forces are generated, thus more sonication energy is applied to the nanoparticle dispersion, which decreases the particle size distribution of the NLCs by breaking larger particles into smaller and monodispersed globules [30].

#### 2.3.2. Effect on PDI (*Y*_2_) 

The PDI of the formulation was an essential aspect that not only determined the particle size distribution and homogeneity of the NLCs formulation but also has a substantial impact on their stability. A smaller PDI value indicates that the particles have a narrow size distribution and are homogeneous in nature. Moreover, a PDI value of under 0.3 is desirable as it indicates a uniform distribution of the particles [31]. Table 3 illustrates the PDI of the TAC-THQ-NLCs obtained from various experimental runs recommended by BBD. Upon considering various experimental runs, it was found that the average PDI ranged from 0.123 to 0.301. The obtained polynomial equation for PDI is expressed as follows:

PDI (*Y*_2_) = +0.1543 + 0.0530 *X*_1_ − 0.0183 *X*_2_ + 0.0323 *X*_3_ − 0.0065 *X*_1_*X*_2_ + 0.0080 *X*_1_*X*_3_ + 0.0025 *X*_2_*X*_3_ + 0.0273 *X*_1_^2^ + 0.0038 *X*_2_^2^ + 0.0223 *X*_3_^2^ (2)

According to the coded quadratic Equation (2) and various plots, as shown in Figure 4, the PDI of the TAC-THQ-NLCs changed when the levels of independent factors were altered. Our findings showed that an increase in the amount of the lipid mixture and sonication time caused the PDI to increase, whereas the concentration of the surfactant had a decreasing effect on the PDI of the NLCs. Upon increasing the concentration of lipids, due to a lack of surfactants, proper emulsification between the lipid phase and aqueous phase did not occur, which eventually led to an increase in PDI. Increasing the surfactant concentration decreased the PDI by reducing the interfacial tension between the oil and water phases, which led to the production of homogeneous particles. Moreover, the effect of the sonication time on the PDI first decreased the PDI up to a certain level, but after that led to the aggregation of the particles, and thus the production of non-uniform particles.

#### 2.3.3. Effect on Entrapment Efficiencies (*Y*_3_ and *Y*_4_) 

Entrapment efficiency is a crucial characteristic in the formulation of NLCs, due to the necessity of delivering the therapeutically effective dose of the drug included in the NLCs. Table 3 illustrates the %EE of the TAC-THQ-NLCs obtained from various experimental runs recommended by BBD. Upon considering various experimental runs, it was found that the average %EE ranged from 58.73 to 83.95% for the TAC and 66.16% to 89.17% for the THQ. The obtained polynomial equation for the %EE of the TAC and THQ is expressed as follows:

%EE-TAC (*Y*_3_) = +71.06 + 9.12 *X*_1_ − 0.7538 *X*_2_ − 2.98 *X*_3_ − 0.6150 *X*_1_*X*_2_ − 0.5200 *X*_1_*X*_3_ + 0.0025 *X*_2_*X*_3_ + 2.13 *X*_1_^2^ + 0.0021 *X*_2_^2^ − 3.27 *X*_3_^2^ (3)

%EE-THQ (*Y*_4_) = +76.75 + 8.11 *X*_1_ − 0.7950 *X*_2_ − 2.56 *X*_3_ − 0.3125 *X*_1_*X*_2_ − 0.0250 *X*_1_*X*_3_ − 0.1475 *X*_2_*X*_3_ + 2.28 *X*_1_^2^ + 0.5683 *X*_2_^2^ − 2.28 *X*_3_^2^ (4)

According to the coded quadratic (Equations (3) and (4)) and the various plots shown in Figure 5 and Figure 6, the %EE of TAC-THQ-NLCs changed when the levels of independent factors were altered. An increase in the concentration of the lipid mixture led to an increase in the %EE of the formulation, as more space was available for the TAC and THQ to become solubilized in the lipid matrix of the NLCs. In addition, adding a liquid lipid (Capryol 90) increased the drug EE by transforming the lipid matrix into an imperfect lattice [32]. On the contrary, the effect of the surfactant concentration has a negative effect on the %EE of the NLCs. Surfactant concentrations, over their critical points, lead to the development of mixed micelles (consisting of drugs and surfactants) that coexist with the lipid nanoparticles and reduce the drug’s entrapment in the nanoparticles [30]. Another reason is that the increase in surfactant concentration leads to the diffusion of drugs from the lipid matrix to the external phase, which eventually leads to a decrease in the entrapment of the drugs in the NLCs [29]. The increase in sonication time showed a decrease in the %EE of the TAC-THQ-NLCs. This may be a result of the increased sonication energy supplied by longer sonication times, which may have triggered NLCs rupture and drug expulsion. 

#### 2.3.4. Selection of Optimized Formulation Composition

The best formulation composition was selected on the basis of set goals i.e., small PS, low PDI, and high %EE. The numerical optimization part of BBD suggested conditions for the TAC-THQ-NLCs preparation as 4.36% lipid-mixture, 6.26% surfactants, and 2.73 min sonication time depending on the applied PS, PDI, and %EE goals, with corresponding predicted value for particle size of 147.98, PDI of 0.164 and %EE of 75.03 and 80.35 for TAC and THQ respectively as shown in Table 5. The optimal level of component and process factors was used to prepare the optimized NLCs. The actual experimental values of PS, PDI, and %EE of the optimized TAC-THQ-NLCs were found to be 144.95 ± 2.80 nm, 0.160 ± 0.021, and 73.44 ± 2.54% and 78.62 ± 2.75% for THQ and TAC, respectively.

### 2.4. Characterization of Optimized TAC-THQ-NLCs 

#### 2.4.1. Particle Characterization

**Figure 7:**
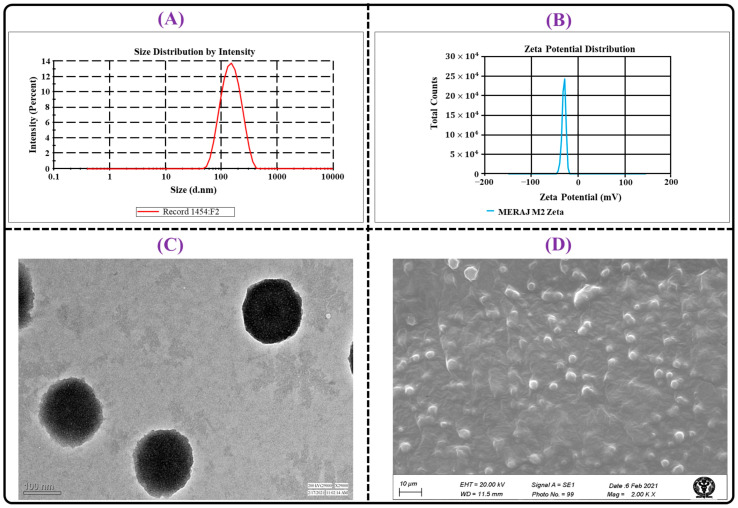
Image illustrating: (**A**) particle size distribution; (**B**) zeta potential distribution; (**C**) TEM photomicrograph; and (**D**) SEM photomicrograph of TAC-THQ-NLCs.

The optimized TC-THQ-NLCs were found to have an average particle size and PDI of 144.95 ± 2.80 nm and 0.160 ± 0.021, respectively, as shown in the representative particle size distribution graph in Figure 7A. The low values of the PS and PDI indicated narrow and homogeneous particle distribution and suitability of the NLCs for skin delivery. It has been claimed that particles below 200 nm enhance skin permeation by ensuring close contact with the SC, which is a major barrier to the delivery of drugs through the skin. Additionally, the fact that the PDI value was below the range of 0.3 indicated that the particle distribution in the NLCs was comparatively homogeneous. The average zeta potential reading for the optimized TAC-THQ-NLCs was found to be −29.47 ± 1.9 mV, as shown in the representative zeta potential distribution graph in Figure 7B. The fact that the TAC-THQ-NLCs had a relatively high zeta potential with a negative value indicated that the formed nanosystem was stable. The negative zeta potential of the developed TAC-THQ-NLCs was observed due to the utilization of Capryol 90 as a liquid lipid in the formulation development. Zeta potential is a critical measure of the degree to which nanoparticles with similar charges will repel one another, which is also correlated to the non-aggregation and physical stability of the nanoparticles (TAC-THQ-NLCs) [33]. 

#### 2.4.2. %EE and %DL 

The %EE of TAC and THQ in TAC-THQ-NLCs was found to be 73.44 ± 2.54 and 78.62 ± 2.75, respectively, showing that optimized TAC-THQ-NLCs, displayed higher entrapment of both drugs at the optimum concentration of lipid matrix. The lipophilicity of the drugs and the imperfect crystal structure of NLCs could be the reason for the high entrapment efficiency of drugs in NLCs, as it leads to higher drug payload capacity [34]. Furthermore, THQ has higher entrapment efficiency than TAC, which could be attributed to their increased solubility in a lipid matrix. The %DL of the TAC and THQ in the optimized TAC-THQ-NLCs were found to be 1.69 ± 0.058 and 5.41 ± 0.191, respectively. 

#### 2.4.3. Morphological Characterization 

The morphological characteristics of optimized TAC-THQ-NLCs were investigated under TEM and SEM, and their representative photographs are illustrated in Figure 7C,D. The shapes were visible in the TEM and SEM micrographs, and were found to be round or oval with a narrow size distribution and to be non-aggregated. The particle size determined by TEM agreed with the DLS data obtained using the zeta-sizer. The optimized TAC-THQ-NLCs 3-dimensional SEM image further supported the surface morphology of the NLCs as being almost spherical with a smooth surface. Thus, these observations showed that a spherical nanosystem was made with a size distribution in the nano-size range (below 200 nm), making it perfect for topical delivery.

#### 2.4.4. FT-IR Analysis 

**Figure 8:**
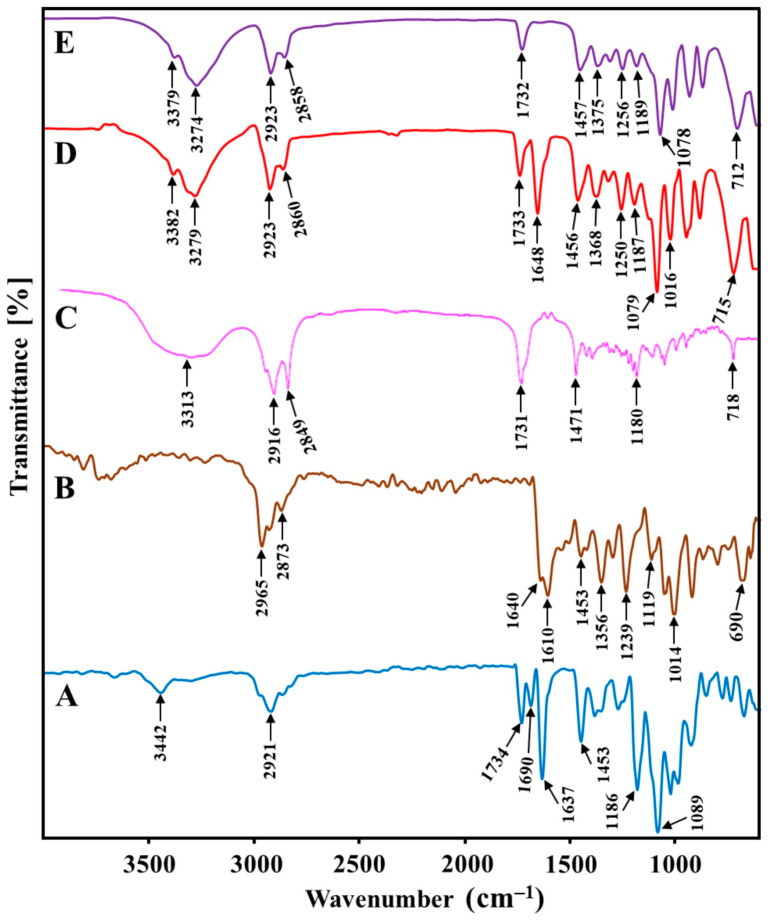
FT-IR spectra of (**A**) TAC; (**B**) THQ; (**C**) GMS; (**D**) physical mixture (TAC, THQ, GMS); and (**E**) TAC-THQ-NLCs.

The FT-IR spectra of the TAC, THQ, GMS, physical mixture (TAC, THQ, and GMS), and optimized TAC-THQ-NLCs were recorded, as depicted in Figure 8, and their distinctive peaks were analyzed for physicochemical interaction between drugs-excipient. The FT-IR spectra of the TAC had characteristic peaks at 3442 (O–H stretching), 2921 (C–H alkanes), 1734 (ester C=O stretching), 1690 (ketonic C=O stretching), 1637 (C=C stretching), 1186 (esteric C=O stretching), 1089 (etheric C=O stretching) cm^−1^ [35]. The FT-IR spectra of the THQ had intense characteristic peaks at 2965, 2873 (C–H stretching), 1640 (C=O stretching), 1610 (C=O, C=C stretching), 1453 (CH_3_ antisymmetric bending), 1356 (CH_3_ symmetric bending) 1239, 1119 and 1014, 690 (C–H bending) cm^−1^ [36]. The FT-IR spectra of the GMS had intense characteristic peaks at 3313 (O–H stretching), 2916, 2849 (C–H stretching), 1731 (C=O stretching), 1471 (CH3 bending), 1180 (C=O bending), and 718 (long chain bands) cm^−1^ [37]. Furthermore, most of the qualitative peaks of TAC and THQ were observed in the FT-IR spectra of the physical mixture (TAC, THQ, and GMS) and TAC-THQ-NLCs. However, the intensity of the peaks was reduced in the final formulation due to the encapsulation of the drugs in the lipid matrix of the NLCs. Since no other characteristic peaks were visible, other than that of drugs and excipients, it can be concluded that no physicochemical interactions took place between the formulation components.

#### 2.4.5. DSC Analysis 

**Figure 9:**
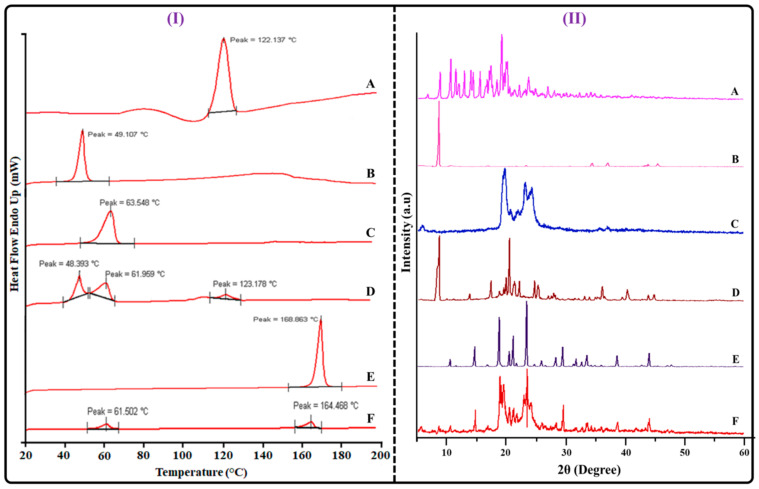
Image showing (**I**) DSC curves (**II**) Powder-XRD of (**A**) TAC; (**B**) THQ; (**C**) GMS (**D**) physical mixture (TAC, THQ, GMS); (**E**) mannitol; and (**F**) lyophilized TAC-THQ-NLCs.

DSC is a crucial tool for the formulation’s solid-state characterization. It provides information regarding the crystalline or amorphous states of the samples as well as the interaction between the formulation’s components. Figure 9I represents the DSC curve of the drugs, excipients, physical mixture (TAC, THQ, GMS), and lyophilized TAC-THQ-NLCs. Pure drugs (TAC and THQ) presented a pronounced endothermic peak at 122.137 °C and 49.107 °C, respectively, which is indicative of their crystalline nature. Similarly, both GMS and mannitol exhibited a pronounced endothermic peak at 63.548 °C and 168.863 °C, respectively. The physical mixture of TAC, THQ, and GMS displayed endothermic peaks at 123.178 °C, 48.393 °C, and 61.959 °C, respectively, indicating that there was no physicochemical interaction between the drugs and excipients included in the TAC-THQ-NLCs. However, the DSC curve of lyophilized TAC-THQ-NLCs showed two endothermic peaks at 61.502 °C and 164.468 °C, which corresponds to GMS and mannitol (used as a cryoprotectant during the lyophilization process), respectively. The disappearance of the endothermic peak of TAC and THQ in the lyophilized TAC-THQ-NLCs indicated that TAC and THQ were completely encapsulated in an amorphous (more stable than crystalline) state within the lipid matrix. Thus, the DSC curves proved conclusively that the drugs were encapsulated in the amorphous form, and also demonstrated the compatibility of the drugs and excipients used in formulating the TAC-THQ-NLCs.

#### 2.4.6. PXRD Analysis 

Following encapsulation in NLCs, the physical state of TAC and THQ was analyzed by PXRD. It determines whether the drugs are present in crystalline or more stable amorphous forms. The diffractogram of TAC, THQ, GMS, physical mixture (TAC, THQ, and GMS), mannitol, and lyophilized TAC-THQ-NLCs are displayed in Figure 9II. The crystallinity of the TAC and THQ was apparent, with sharp and distinct two-theta (2θ) values between 8 to 25 °C for the TAC and a characteristic sharp peak at 8.52 °C for the THQ. The waxy nature of the GMS was demonstrated by a high-intensity peak in the diffractogram of the GMS at 2θ value equivalent to 19.32, 19.64, and 23.0, 24.18 °C. In the physical mixture, the crystalline peaks for TAC, THQ, and GMS were evident at almost the same 2θ value. In contrast, the TAC-THQ-NLCs formulation showed a broad deformed peak and the absence of prominent peaks of TAC and THQ, demonstrating encapsulation of drugs within the lipid matrix of NLCs in an amorphous form. However, individual diffractograms of the mannitol (cryoprotectant used during lyophilization) and GMS further confirmed that the peaks in the NLCs were due to the presence of excipients.

### 2.5. Formulation and Characterization of TAC-THQ-NG and TAC-THQ-SG

#### 2.5.1. Physical Appearance, pH, and Drug Content 

**Figure 10:**
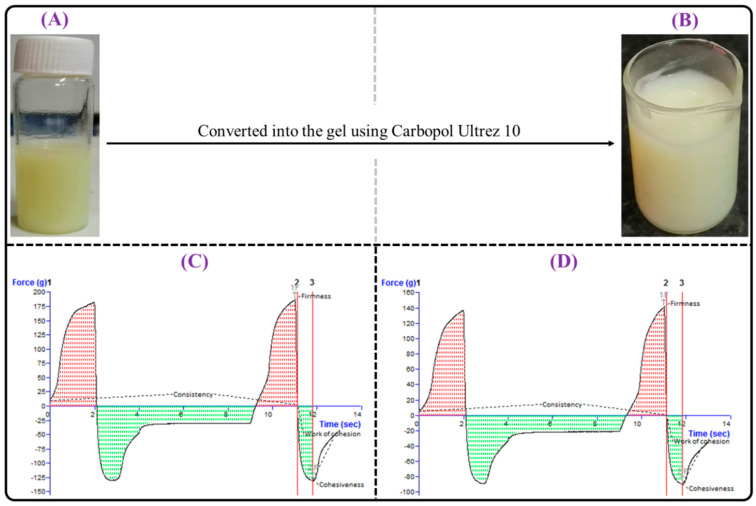
Image showing physical appearance of: (**A**) TAC-THQ-NLCs; and (**B**) TAC-THQ-NG. Representative texture analysis graph of (**C**) TAC-THQ-NG; and (**D**) TAC-THQ-SG showing firmness, consistency, cohesiveness, and work of cohesion.

Figure 10A,B shows the physical appearance of TAC-THQ-NLCs and TAC-THQ-NG. The TAC-THQ-NLCs was pale yellow, free from aggregation, and homogeneously dispersed. The TAC-THQ-NG had an aesthetically pleasing and consistent appearance with light yellowish color and the absence of any rough particles. The pH values of the TAC-THQ-NG and TAC-THQ-SG were shown to be 6.72 ± 0.29 and 6.80 ± 0.38, respectively, both of which are safe and compatible with skin in order to eliminate the chance of skin irritation following application. The drug content in the TAC-THQ-NG was found to be 97.99 ± 1.79% and 98.51 ± 1.33% for TAC and THQ, respectively. The contents of TAC and THQ in the TAC-THQ-SG formulation were found to be 98.19 ± 1.49% and 99.05 ± 1.26%, respectively. In both formulations, TAC and THQ contents were within acceptable limits and were evenly distributed throughout the formulation. 

#### 2.5.2. Viscosity and Spreadability 

The viscosity and spreadability of TAC-THQ-NG were measured so that a suitable pharmaceutical composition could be made that was consistent, stable, and easy to use. The viscosity was found to be 2.33 ± 0.35 and 2.12 ± 0.28 Pa·s for TAC-THQ-NG and TAC-THQ-SG, respectively, which represents the excellent physical behaviour of the gel. The high viscosity enhances the gel’s residence time on the skin surface, resulting in an increased permeation time. From the perspective of patient compliance, spreadability is a crucial component of topical formulations. The formulation is more comfortable to apply to an inflamed area if the base spreads easily on the skin. The final diameter was found to be 8.10 ± 0.46 cm and 7.23 ± 0.40 cm, respectively, for the TAC-THQ-NG and TAC-THQ-SG, which is indicative of good spreadability. However, the TAC-THQ-SG had lower viscosity and spreadability than TAC-THQ-NG, which might be related to the formulation’s components [24]. 

#### 2.5.3. Texture Analysis 

The peak force is a measurement of the firmness of the formulation and is indicative of gel strength. The positive area of the curve up to this point measures consistency and it is suggestive of the spreadability of the gel. The maximum negative force indicates the cohesiveness of the sample and is indicative of the extrudability of the sample from the tube. The negative area of the curve is called the ‘work of cohesion’, and it indicates the cohesiveness and consistency/viscosity of the formulation [38]. The force–time curves of TAC-THQ-NG and TAC-THQ-SG obtained from the texture analyzer are shown in Figure 10C,D. It was found that the optimized NLC-gel, as depicted in Figure 10C, showed slightly higher firmness (186.44 g), consistency (137.22 g·s), cohesiveness (−131.04 g), and work of cohesion/index of viscosity (−73.40 g·s) as compared to the placebo gel, which had the following values: firmness (141.53 g), consistency (84.37 g·s), cohesiveness (−89.60 g), and work of cohesion/index of viscosity (−50.40 g·s), as shown in Figure 10D. The possible reason may be the presence of excipients in the NLCs formulation. The above-found values mainly suggest the easy skin-application capacity of the produced TAC-THQ-NG and TAC-THQ-SG [39]. 

### 2.6. In Vitro Drug Release and Release Kinetics 

**Figure 11:**
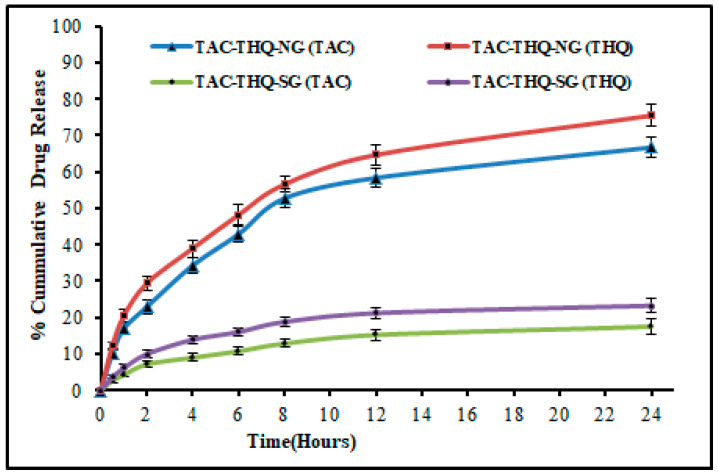
Comparative in vitro drug-release profiles of TAC and THQ from the optimized TAC-THQ-NG and TAC-THQ-SG.

The cumulative percentage releases of TAC and THQ from TAC-THQ-NG and TAC-THQ-SG were determined in vitro using a dialysis bag over a period of 24 h. The mean percentages of the cumulative drug release of TAC-THQ-NG and TAC-THQ-SG formulations were evaluated and their respective release profiles are shown in Figure 11. TAC-THQ-NG demonstrated a biphasic release pattern with initial burst release followed by comparatively sustained drug release with 66.70 ± 2.80% and 75.42 ± 2.97% of TAC and THQ released, respectively, at 24 h. The initial burst release may have been caused by a certain amount of TAC and THQ precipitating from the superficial lipid matrix or being adsorbed over the NLCs surface. However, the sustained drug release from TAC-THQ-NG suggests that the TAC and THQ were diffused from the lipid matrix core of the NLCs out into the release medium. On the other hand, the TAC and THQ releases from the TAC-THQ-SG were slow with only 17.43 ± 1.99% and 23.20 ± 1.94%, respectively, within 24 h, due to the hydrophobic nature of TAC and THQ. Aside from that, THQ (log P of 2.2 and MW of 164.201 g/mol) release from the formulations was higher than that of the TAC (log P of 3.3 and MW of 822 g/mol), due to the lower MW and lower lipophilicity as compared to TAC [40]. 

On fitting the in vitro drug release data into different release kinetic models, as shown in Table 6, the Korsmeyer–Peppas model was followed with a higher R^2^ value of 0.9892 for TAC, and 0.9822 for THQ from TAC-THQ-NG. Since both TAC and THQ were found to have release exponents (n) of less than 0.5 from TAC-THQ-NG, this is indicative of a Fickian diffusional mechanism for their release.

### 2.7. MTT Assay

**Figure 12:**
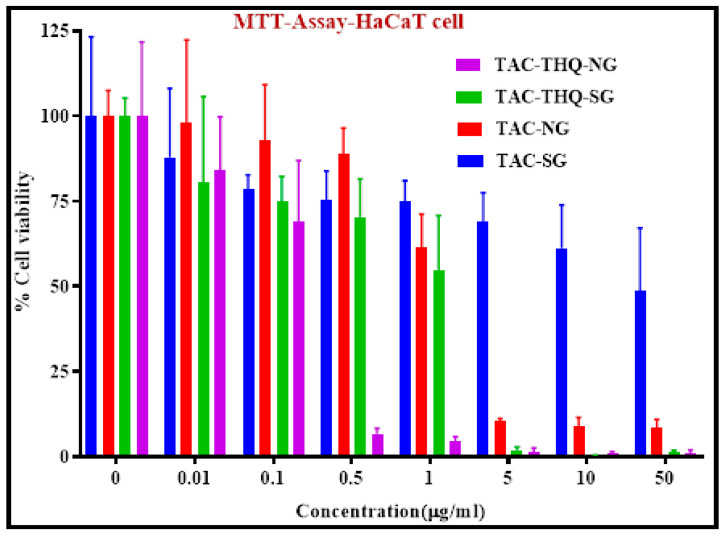
Anti-proliferation effects of TAC-SG, TAC-NG, TAC-THQ-SG, and TAC-THQ-NG against HaCaT cells after an incubation period of 24 h.

Psoriasis is characterized primarily by epidermal overexpression with keratinocyte hyperproliferation and differentiation in the epidermis, which produces silvery-scale lesions. Inhibiting epidermal cell proliferation is essential for achieving a more effective therapeutic result in treating psoriasis. Therefore, an MTT assay was performed at 24 h to evaluate the antiproliferative effect of different formulations (TAC-THQ-NG, TAC-THQ-SG, TAC-NG, and TAC-SG) on HaCaT cell proliferation, which are depicted in Figure 12. According to the results, all formulations reduced the viability of HaCaT cells in a concentration-dependent manner. Interestingly, the HaCaT cell viability decreased significantly in the order of TAC-THQ-NG < TAC-THQ-SG< TAC-NG < TAC-SG at 24 h, respectively. The IC_50_ values were 0.1303 μgmL^−1^, 0.876 μgmL^−1^, 1.528 μgmL^−1^, and 18.93 μgmL^−1^, for TAC-THQ-NG, TAC-THQ-SG, TAC-NG, and TAC-SG, respectively, at 24 h of study. The IC_50_ values showed a higher reduction of hyperproliferative epidermal cells by TAC-THQ-NG compared with that of the TAC-THQ-SG. Compared to free drugs, the increased uptake of the NLCs formulation is responsible for their higher ability to suppress cell proliferation [41]. Here, TAC-NG and TAC-SG formulations containing a single drug showed lower inhibition of cell viability than TAC-THQ-NG and TAC-THQ-SG containing dual drugs, confirming the enhanced cytotoxicity against HaCaT cell of both the TAC and THQ. According to these findings, using TAC-THQ-NG as a treatment for psoriasis might be more effective than using free drugs.

### 2.8. Antioxidant Activity

**Figure 13:**
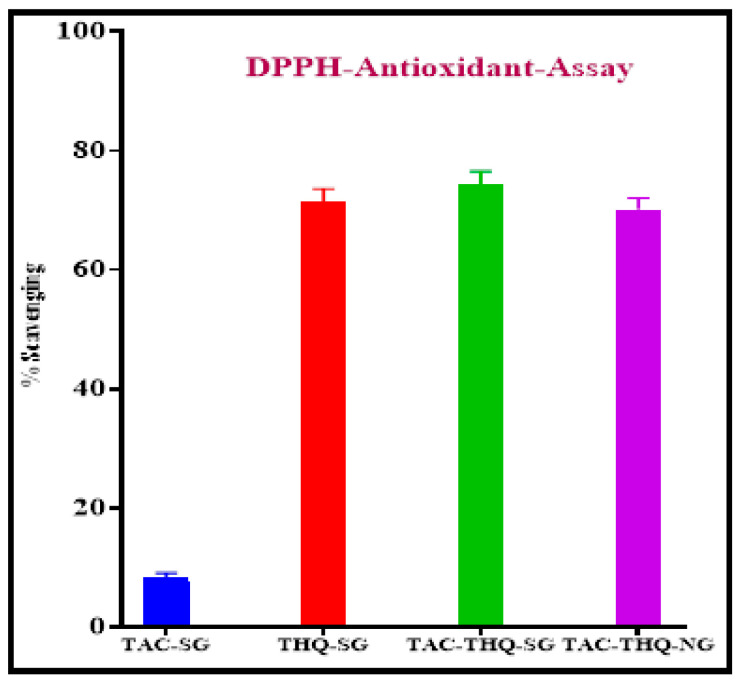
In vitro antioxidant activities of (**A**) TAC-SG; (**B**) THQ-SG; (**C**) TAC-THQ-SG; and (**D**) TAC-THQ-NG.

There is compelling evidence that free radicals, reactive oxygen species (ROS), the compromised function of antioxidants, and oxidative stress are responsible for the pathophysiology of psoriasis [42]. Therefore, the use of drugs having suitable antioxidant activity and retaining their antioxidant potential after encapsulation into the formulation is a prerequisite. Therefore THQ, an herbal drug having antioxidant potential, was used in combination with TAC. The percentages of the free radical scavenging activity of TAC-SG, THQ-SG, TAC-THQ-SG, and TAC-THQ-NG are depicted in Figure 13. Pure THQ showed 71.44 ± 2.17% free radical scavenging as compared to 74.31 ± 2.26% and 70.11 ± 1.97% for TAC-THQ-SG and TAC-THQ-NG, respectively. THQ showed strong antioxidant activity compared to pure TAC, which showed negligible free-radical-scavenging activity against DPPH. Additionally, THQ’s ability to scavenge free radicals was retained even after being combined with TAC in free form and after being encapsulated in the NLCs with TAC.

### 2.9. Assessment of Permeation Depth of the Formulation in the Skin

**Figure 14:**
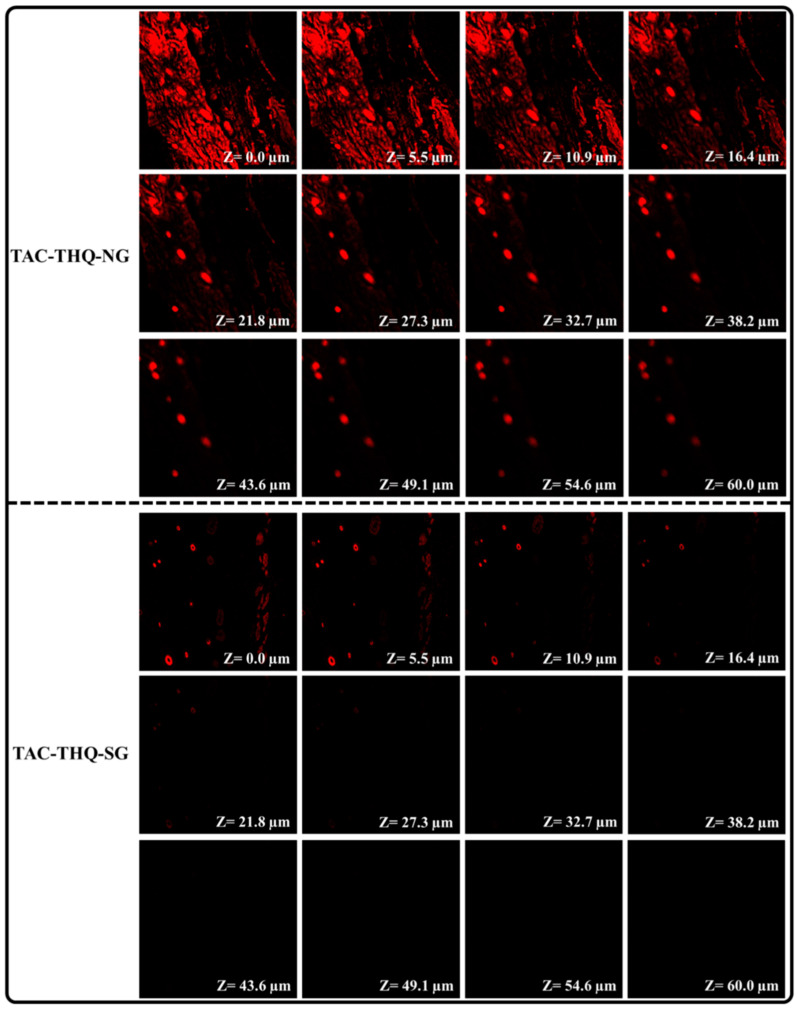
Confocal images of the perpendicular cross-section of mice skin treated with TAC-THQ-NG and TAC-THQ-SG.

The skin distribution of the TAC-THQ-NG and TAC-THQ-SG was assessed using confocal laser microscopy. Figure 14 depicted the CLSM images of mice skin treated with TAC-THQ-NG and TAC-THQ-SG. As shown in Figure 14, it is clearly visible that TAC-THQ-SG could not penetrate deeper skin layers and was limited to a depth of 21.8 μm. As illustrated in Figure 14, the optimized formulation (TAC-THQ-NG) exhibited more significant penetration potential and fluoresced beyond 60 μm. Furthermore, it is evident from the fluorescence intensity of the CLSM image that the TAC-THQ-NG formulation was consistently dispersed throughout the viable layers of the skin, resulting in increased deposition of TAC and THQ between the epidermal and dermal layers of the skin, which are prone to psoriasis. Apart from this, Tween 80, used in the TAC-TAC-NG formulation as a surfactant, is a proven permeation enhancer; therefore, and aids in overcoming the SC, which prevents drugs from penetrating through the scaly psoriatic skin layer [43].

### 2.10. Storage Stability

The stability data of TAC-THQ-NLCs and TAC-THQ-NG stored at different storage conditions for a period of 3 months are shown in Table 7. TAC-THQ-NLCs formulation stored at refrigerated condition (5 ± 3 °C) and room temperature (25 ± 2 °C) showed no substantial shifts in PS, PDI, and EE (TAC and THQ) after a period of 3 months. The developed TAC-THQ-NG showed insignificant changes in drug content (TAC and THQ), pH, and spreadability when stored in refrigerated conditions and at room temperature. The results indicated that the TAC-THQ-NLCs and TAC-THQ-NG are stable for up to three months. Therefore, it is recommended that TAC-THQNLCs or TAC-THQ-NG be stored at a lower temperature to maintain the quality attributes, such as PS, PDI, EE, drug content and pH, and spreadability, of the formulation.

## 3. Conclusions

Psoriasis is a chronic skin condition that impacts patients both physically and socially. Patients need to undergo treatment on a regular basis for many years to avoid recurrence. Monotherapies often fail to treat the disease effectively. Hence, it is very important to deliver multiple drugs to the inner-skin tissues. Here, TAC-THQ-NLCs were formulated using an emulsification solvent-evaporation technique and optimized using 3^3^-BBD. The optimized TAC-THQ-NLCs showed nanometric-particle-size distribution with good entrapment efficiency. An in vitro anti-proliferation study showed a dose-dependent effect on the HaCaT cells (keratinocyte line) with TAC-THQ-NGs showing higher efficacy than their suspensions. An in vitro drug release study showed a sustained release of TAC and THQ for 24 h from the TAC-THQ-NG. Moreover, the depth of skin permeation measured by CLSM revealed higher permeation of the TAC-THQ-NG as compared to that of the TAC-THQ-SG. The above results proved the potential of the optimized TAC-THQ-NLCs for not only improving the treatment of psoriasis but at the same time reducing dose-related toxicity, which needs to be validated by conducting in vivo studies.

## 4. Materials and Methods

### 4.1. Materials and Chemicals

Tacrolimus (TAC) was received as a gift sample from Concord Biotech, Gujarat, India. Thymoquinone (THQ) and Rhodamine B were purchased from Sigma Aldrich, Saint Louis, MO, USA. Capryol 90, Labrasol, Gelot 64, Apifil CG, Precirol ATO 5, Labrafil M 1944 CS, Lauroglycol 90, Peceol, Masine 35-1, and Plurol Diisostearique were obtained from Gattefose India Pvt. Ltd., Mumbai, India as a gift sample. Triethanolamine, stearic acid and glyceryl monostearate (GMS; also called monostearin) were procured from CDH Fine Chemicals (New Delhi, India). (Span 80 and 20), Mannitol and Tween 80 were purchased from SD Fine Chemicals (Mumbai, India). PEG 400 was procured from Thomas Baker Chemicals, New Delhi, India. Lubrizol India (Mumbai, India) was kind enough to gift the Carbopol Ultrez 10 NF for this work. Analytical-grade acetonitrile and methanol were procured from Qualigens Fine Chemicals, Mumbai, India. The remaining chemicals and reagents used for this research work were of analytical grade.

### 4.2. Estimation of TAC and THQ via RP-HPLC

The concentration of TAC and THQ in the developed NLCs was simultaneously measured by our developed RP-HPLC (Shimadzu, SPD-M20A, Tokyo, Japan) technique. For chromatographic separation of drugs, RP-HPLC apparatus with a C-18 column (Shimadzu, 250 × 4.6 mm dimension) was used, equipped with a photodiode array (PDA) detector. Acetonitrile and water were used in binary combination as the mobile phase; at a flow rate and ratio of 0.6 mL/min and 90:10, *v*/*v*, respectively. The sample volume was kept at 20 μL with a total run time fixed for this elution process at 20 min. Prior to analysis, the samples were degassed and passed through a (0.2 µ) nylon filter. Finally, the concentrations of TAC and THQ were measured at wavelengths of 210 nm and 254 nm, respectively.

### 4.3. Excipients Screening

#### 4.3.1. Screening of Liquid Lipid, Solid Lipid, and Binary Mixture

For developing the TAC-THQ-NLCs, the lipids were selected based on the saturation solubility of drugs. For selecting the liquid lipid (oil), different liquid lipids in 1 mL quantities were transferred into micro-centrifuge tubes (MCT), and an excess quantity of the drug was gradually added and dissolved using a mechanical shaker (Remi Elektrotechnik Ltd., Mumbai, India) at room temperature for 24 h. Then, the samples were centrifuged for 0.5 h at 5000 rpm at room temperature (Remi Elektrotechnik Ltd., Mumbai, India). Thereafter, the supernatant from each tube was collected and dissolved in methanol, filtered with a membrane filter (0.2 µ), and the saturation solubility of the drug was quantified spectrophotometrically (UV-1601, Shimadzu, Japan) [44]. For selecting solid lipids, different solid lipids in 500 mg quantities were transferred into 5 mL glass vials and then heated (5 °C) above their melting points. After that, the drugs were added gradually to the melted lipids until saturation solubility was achieved and assessed visually [44,45]. Before selecting the lipid mixture (mixture of solid and liquid lipids), the compatibility of the binary mixture was examined by mixing them with each other and analyzing them visually. The lipids (solid and liquid) with the highest solubility and best compatibility were mixed in different proportions (from 90:10 to 10:90) with continuous stirring at 200 rpm, maintaining the temperature 5 °C above the melting points. The binary mixture that did not show phase separation for 24 h and revealed better miscibility and compatibility was chosen for the development of the TAC-THQ-NLCs [46].

#### 4.3.2. Screening of Surfactants and Co-Surfactants

For the successful development of the NLCs, the surfactants were selected based on their emulsification capacity of lipid mixture. Hence, for selecting the best surfactant, a lipid mixture (100 mg) was dissolved in 3 mL dichloromethane, and the aqueous solution of different surfactants solution (10 mL; 5% *v*/*v*) was added into the organic phase containing lipids mixture under constant stirring. Next, the organic solution was evaporated by heating at 40 °C for 3–4 h on a digital magnetic stirrer. The resulting solution was further diluted 10 times using Milli-Q water, and the %transmittance was noted spectrophotometrically at 510 nm wavelength. The surfactant which revealed the highest %transmittance was considered the best for developing the NLCs [47]. Since the required hydrophilic–lipophilic balance (rHLB) also plays a vital role in developing NLCs with stable, small-sized particles, better homogeneity, and high encapsulation efficiency; therefore, the rHLB of the surfactants was also taken into consideration while selecting surfactants for the development of the NLCs [48].

### 4.4. Formulation of TAC-THQ-NLCs

Tacrolimus and thymoquinone co-loaded NLCs (TAC-THQ-NLCs) were prepared using the previously described emulsification solvent-evaporation technique with slight modifications [49,50]. Briefly, the organic phase was prepared by dissolving Capryol 90 (liquid lipid), GMS (Solid lipid), TAC, and THQ in dichloromethane (DCM). Separately, the mixtures of surfactant and co-surfactant (i.e., Tween 80 and Span 20) were dissolved in 20 mL of double-distilled water to prepare the aqueous phase. Thereafter, the organic phase was added dropwise by using a syringe into the aqueous phase under a continuous stirring speed of 1000 rpm on a digital magnetic stirrer (Remi Elektrotechnik Ltd., Mumbai, India) and then subjected to ultrasonication using a probe-type sonicator (Hielscher, UP-50H, Stahnsdorf, Germany). Finally, the emulsion was stirred at 300 rpm for 8 h to evaporate the organic phase to obtain the final NLCs formulation. The optimized TAC-THQ-NLCs was further lyophilized for solid-state characterization by adding 5% *w*/*v* mannitol as a cryoprotectant.

### 4.5. Experimental Design for Optimization of TAC-THQ-NLCs

For the development of NLCs with desired physicochemical characteristics, three factors (lipid mixture, surfactant mixture, and sonication time) and three levels (particle size, polydispersity index, and entrapment efficiency) based on the Box–Behnken design were selected for the optimization of our developed formulation using Design-Expert^®^ software V.11.0 (State-Ease Inc., Minneapolis, Minnesota, USA). For the optimization study, different codes were assigned for the independent as well as the dependent factors. Codes for the independent factors were assigned as follows: “*X*_1_” for lipid mixture (3–5% *w*/*v*), “*X*_2_” for surfactant mixture (5–7% *w*/*v*), and “*X*_3_” for sonication time (2–4 min.) while codes for dependent variables were chosen as: “*Y*_1_” for particle size (nm), “*Y*_2_” for PDI, “*Y*_3_” and “*Y*_4_” for entrapment efficiency of TAC and THQ (%EE). The above-mentioned parameters with their respective values are summarized in Table 8. The values of factors were taken at three levels: high (“+1”), medium (“0”), and low (“−1”). The 3^3^-BBD provides 15 different combinations, including 3-center points (repetitions) for the development of the NLCs. Each formulation, as per the composition, was prepared to obtain the results, which were later fitted into the design as given in Table 3. Thereafter, the data were analyzed with different statistical models, i.e., linear, 2-FI, cubic, and quadratic, by one-way analysis of variance (ANOVA). The model that represented the highest overall desirability value was considered to be the ideal model. Moreover, the selected model was used for the generation of polynomial equations and different response plots, which were later analyzed for the understanding of the relationship between independent and dependent variables [51].

### 4.6. Characterization of TAC-THQ-NLCs

#### 4.6.1. Particle Characterization

The particle size (PS), polydispersity index (PDI), and zeta potential (ZP) of the developed TAC-THQ-NLCs were measured by a zeta sizer (Nano-ZS, Malvern Instruments, Worcestershire, UK) by the principle of dynamic light scattering. For analysis, the formulations were diluted 20 times with deionized water and then analyzed at a 90° angle of scattering at room temperature. Similarly, for the determination of ZP, the same samples were placed in the electrophoretic cell before analysis [52]. All the analyses were performed in triplicate, while the results were documented as mean ± SD.

#### 4.6.2. Entrapment Efficiency (%EE) and Drug Loading (%DL)

The %EE and %DL of the TAC-THQ-NLCs were determined indirectly by the ultracentrifugation technique. Briefly, the formulations were transferred to a centrifuge tube and subjected to high-speed centrifugation (Sigma 3K30, Sigma Laborzentrifugen GmbH, Osterode am Harz, Germany) at 15,000 rpm for 15 min. After that, the supernatant was collected, diluted appropriately with methanol, and filtered with a 0.2 µ membrane filter. After that, the concentration of unencapsulated drugs was quantified by RP-HPLC [53]. Finally, the %EE and %DL were calculated by the following formulae.

(5) $$\%\mathrm{EE}=\frac{\mathrm{Total}\;\mathrm{drug}-\mathrm{Unencapsulated}\;\mathrm{drug}}{\mathrm{Total}\;\mathrm{drug}}\times 100$$

(6) $$\%\mathrm{DL}=\frac{\mathrm{Total}\;\mathrm{drug}-\mathrm{Unencapsulated}\;\mathrm{drug}}{\mathrm{Total}\;\mathrm{weight}\;\mathrm{of}\;\mathrm{NLCs}}\times 100\;$$

#### 4.6.3. Morphological Characterization

The shape and surface morphology of the optimized TAC-THQ-NLCs were characterized with the help of an electron microscope utilizing an instrument, transmission electron microscope (TEM), and scanning electron microscopy (SEM). For TEM analysis, one drop of 20 times diluted optimized formulation was mounted on a copper grid coated with copper. Thereafter, the formulation-bearing grid was stained negatively with phosphotungstic acid (2% *w*/*v*) to enhance the contrast. Finally, the sample was air-dried at room temperature before being examined under TEM (HR-TEM, Fei, Electron Optics, Netherlands) operated at 200 kV with point-to-point resolution. For SEM analysis, 20 µL of the formulation was placed on the aluminum stub-bearing double-sided tape. Thereafter, the formulation-bearing stub was subjected to sputter coating with a mixture of platinum and palladium until a thickness of 5 nm was achieved to improve overall conductivity. Finally, the sample was visualized under SEM (SEM, EVO 18 model, Zeiss, Germany) operated at 20 kV voltage.

#### 4.6.4. Fourier-Transform Infrared (FT-IR) Spectroscopy

FT-IR spectroscopy was utilized to look for any notable physicochemical interactions between the drugs and the excipients used in the formulation of the TAC-THQ-NLCs. Therefore, the FT-IR spectra of TAC, THQ, GMS, a physical mixture (TAC, THQ, and GMS), and TAC-THQ-NLCs were recorded by utilizing the FT-IR instrument (ATR mode; Tensor 37, Bruker, Germany). The FT-IR spectrum of each sample was taken from the wavelength ranging from 4000 cm^−1^ to 400 cm^−1^.

#### 4.6.5. Differential Scanning Calorimetry (DSC)

The DSC curves of drugs (TAC and THQ), physical mixture (TAC, THQ, and GMS), and lyophilized TAC-THQ-NLCs were recorded using a differential scanning calorimeter (DSC; Perkin Elmer, Waltham, MA, USA) to investigate the compatibility between the drugs and excipients, as well as the physical state of the drug within the lipid matrix of the NLCs. For conducting the DSC analysis, powder samples (2 mg) were taken into the aluminum pan and sealed. Then, the DSC curves of each sample were recorded from temperatures ranging between 20 °C to 200 °C. The temperature was calibrated by using indium (>99.95% purity) with a melting point of 156.6 °C. An empty aluminum pan was used as a reference. The nitrogen gas flow and rate of heating were set to 20 mL/min and 10 °C/min, respectively.

#### 4.6.6. Powder X-ray Diffraction (PXRD)

An X-ray diffraction study was conducted to examine the crystalline or amorphous characteristics of drugs encapsulated in the matrix of the NLCs. The XRD diffractograms of TAC, THQ, GMS, physical mixture (TAC, THQ, GMS), mannitol, and lyophilized TAC-THQ-NLCs were recorded using an X-ray diffractometer (D8-Advanced, Bruker, AXS, Karlsruhe, Germany). The samples were kept inside the sample holder of the XRD instrument, and the graph was recorded as 2θ values, ranging from 5° to 60° at a 10° scanning speed.

### 4.7. Preparation of TAC-THQ-NLC-Gel and TAC-THQ-Suspension-Gel

The application of TAC-THQ-NLCs on the skin is difficult due to their insufficient viscosity. Therefore, the optimized TAC-THQ-NLCs were converted into gels with the addition of a gelling agent (Carbopol Ultrez 10; 1% *w*/*w*) under constant stirring, placed on a magnetic stirrer maintained at 1000 rpm until the gelling agent fully dispersed. Thereafter, the resulting mixture was left overnight for swelling, followed by neutralization with a triethanolamine addition (0.05% *w*/*w*) added dropwise to convert it into a TAC-THQ-NLC-gel (TAC-THQ-NG). A similar procedure was followed to prepare the TAC-THQ-suspension-gel (TAC-THQ-SG) [25].

### 4.8. Characterization of TAC-THQ-NG and TAC-THQ-SG

#### 4.8.1. Physical Appearance, pH, and Drug Content

The physical appearance, as well as the homogeneity of the prepared gels, was examined visually. The gel was analyzed for its pH using a pH meter (Mettler Toledo MP 220, Greifensee, Switzerland) at room temperature. The drug content was calculated to assess the uniformity of drugs present in the prepared gels. For determining the TAC and THQ content in gels, 1 g of gels were dissolved in 10 mL methanol, filtered with 0.2 µ membrane filter, diluted appropriately, and then subjected to RP-HPLC [54].

#### 4.8.2. Viscosity and Spreadability

The viscosity of the optimized TAC-THQ-NG and TAC-THQ-SG were determined with the help of a Brookfield viscometer (Brookfield Laboratories, Middleboro, MA, USA). The instrument was operated at 100 rpm for 160 s at room temperature [55]. The spreadability was evaluated by positioning 500 mg of gels within a radius of 0.5 cm (that is, within a 1 cm diameter) of a pre-marked circle on a glass plate and then placing a second glass plate on top of the first glass plate. Next, 500 g weight was applied to the upper portion of the glass plate for 5 min, and an increment in diameter was noted. The gel’s spreadability as a weight function was assessed by measuring the increments in the diameter [56].

#### 4.8.3. Texture Analysis

The texture analysis of the developed TAC-THQ-NG and TAC-THQ-SG were performed using the instrument TA.XTplus Texture Analyzer (Stable Micro Systems, Surrey, UK) at room temperature. Briefly, 100 g of each gel formulation (TAC-THQ-NG and TAC-THQ-SG) was placed into a 100 mL glass beaker and the surface was kept flat to avoid forming air bubbles. After that, different parameters of the gel, such as firmness, consistency, cohesiveness, and cohesion, were determined using the texture analyser software and represented in the form of a graph [57].

### 4.9. In Vitro Drug Release and Release Kinetics

A dialysis bag (MWCO: 12 kDa; Sigma-Aldrich, USA) technique was followed to investigate the TAC and THQ release from the TAC-THQ-NG and TAC-THQ-SG. The dissolution media were prepared by mixing 0.5% *w*/*v* Tween 80 with the (PBS; pH 7.4) phosphate buffer saline. For this study, the release media (200 mL) were taken into a vessel, in which the dialysis bag containing 1 gm (TAC-THQ-NG) and (TAC-THQ-SG) of the gel formulations were immersed. This assembly was positioned on a magnetic stirrer with a temperature of the plate regulated at 37 ± 1 °C and 100 rpm speed. The duration of the study was 24 h, and at every pre-decided time point, a sample volume of 2 mL was taken out, and the same volume of the fresh dissolution media was replaced. Thereafter, withdrawn samples were passed through a 0.2 μ membrane filter before being analyzed using RP-HPLC for drug quantification. The data obtained were subjected to different kinetics models to determine the drug release mechanism from the TAC-THQ-NG [58].

### 4.10. MTT Assay

The antiproliferative activity of the TAC-THQ-NG, TAC-THQ-SG, TAC-NLC-gel (TAC-NG), and TAC-suspension-gel (TAC-SG) was evaluated in vitro using an MTT assay on the HaCaT cells (immortalized human epidermal cells). For this assay, 1 × 10^5^ HaCaT cells per well were transferred for culturing into 96 well plates and placed in a CO_2_ incubator for 24 h. The culture media consisted of a DMEM medium containing fetal bovine serum (FBS; 10%) and antibiotic solution (Penicillin; 1%), and the temperature was maintained at 37 °C with CO_2_ (5%). Post incubation, various samples prepared at different concentrations (0.01–50 µg/mL) were added to the HaCaT cells and kept for incubation for 24 h. Subsequently, MTT dye at 250 µg/mL was mixed well and further subjected to incubation for 2 h. After that, the culture media were removed from each well, followed by the addition of 100 µL DMSO to solubilize the developed formazan crystals. Next, the plate was placed for 5 min on a shaker to solubilize the formazan crystals completely. Finally, absorbance at 570 nm wavelength was noted using a microplate reader (iMark, Biorad, USA). Cell viability was calculated from the absorbance data, and the results are reported as their corresponding IC_50_ values.

### 4.11. Antioxidant Activity

The 2, 2-diphenyl-1-picrylhydrazyl (DPPH) assay method was used for the estimation of the antioxidant activity of the optimized formulation [59]. Briefly, the stock solutions of TAC-SG, THQ-SG, TAC-THQ-SG, and TAC-THQ-NG (equivalent to 0.1 mg TAC and 0.3 mg THQ) were prepared by dissolving in methanol. Then a methanolic solution of the pure drug(s) in gel and those extracted from the NLC gel were mixed with an equal volume of 0.3 mM DPPH solution. The resulting mixture was shaken to complete the reaction and stored for 30 min. at room temperature in a dark place. The absorbance was noted at 517 nm spectrophotometrically (Shimadzu Corp, Kyoto, Japan). Finally, the antioxidant activity was calculated by the given formula:

(7) $$\mathrm{Antioxidant}\;\mathrm{activity}\;\left(\%\right)=1-\frac{{\mathrm{Abs}}_{\mathrm{Sample}}}{{\mathrm{Abs}}_{\mathrm{Control}}}\times 100$$

where ${\mathrm{Abs}}_{\mathrm{Sample}}$ and ${\mathrm{Abs}}_{\mathrm{Control}}$ refer to the absorbance of the sample and DPPH solution (control), respectively.

### 4.12. Assessment of Permeation Depth of the Formulation in the Skin

The depth of the permeated formulations (TAC-THQ-NG and the TAC-THQ-SG) in the skin was analyzed by confocal laser scanning microscopy (CLSM). For conducting this experiment, the skin (1 cm^2^ dimension) was excised, hairs and subcutaneous fat were cleaned and then washed at least 3 times with phosphate buffer saline (PBS; pH 7.4) and fixed on the Franz diffusion cell between the donor and acceptor compartment. In this experiment, PBS; pH 7.4, 0.5% Tween 80 were taken as the diffusion media; hence the receptor compartment was filled with 10 mL PBS. After that, Rhodamine B (0.03%)-loaded NLC-gel and pure Rhodamine B-based gel were spread separately on the abdominal skin of mice fixed on Franz diffusion cells. This assembly was placed on the top of an electronic magnetic stirrer fixed at 100 rpm speed for 24 h, maintaining the temperature at 37 ± 1 °C. Thereafter, the skin was taken and washed with ethanol, and slides of 0.5 µm thickness were prepared. Finally, the permeation depths of both samples were visualized under a confocal microscope (CLSM; Leica Microsystems, Wetzlar, Germany) [17,60].

### 4.13. Storage Stability

The physicochemical stability of the TAC-THQ-NLCs and TAC-THQ-NG was evaluated at three different storage conditions i.e., 5 ± 3 °C (refrigerated temperature) and 25 ± 2 °C (room temperature) for a period of 3 months. The samples were cautiously transferred to a glass vial and placed for 3 months under different storage conditions. After 3 months, samples were evaluated for PS, PDI, and %EE for the TAC-THQ-NLCs whereas TAC-THQ-NG formulations were evaluated for changes in pH, drug content, and spreadability [61].

### 4.14. Statistical Analysis

All experimental data are presented as mean ± standard deviation (SD), and GraphPad Prism 8 software version 8.3.0 was used for statistical analysis. One-way ANOVA was used to determine the level of significance of the data that was acquired, and then Tukey’s multiple comparison tests were used. A statistically significant difference was defined as *p* < 0.05.

## Figures and Tables

**Table 1:** Screening of binary lipid-mixture ratio based on miscibility.

Solid Liquid	Liquid Lipid	Ratio (S:L)	Inference
GMS	Capryol 90	1:9	Phase separation
GMS	Capryol 90	2:8	Phase separation
GMS	Capryol 90	3:7	Phase separation
GMS	Capryol 90	4:6	Phase separation
GMS	Capryol 90	5:5	Phase separation
GMS	Capryol 90	6:4	No phase separation
GMS	Capryol 90	7:3	No phase separation
GMS	Capryol 90	8:2	No phase separation
GMS	Capryol 90	9:1	No phase separation

**Table 2:** % Transmittance of Binary lipid mixture by various surfactants.

Surfactants	% Transmittance ± SD
Tween 80	91.03 ± 2.82
Span 80	77.96 ± 2.59
PEG 400	86.03 ± 2.42
Lauroglycol 90	81.06 ± 2.95
Plurol Diisostearique	82.93 ± 2.22
Labrasol	89.30 ± 3.22
Span 20	86.77 ± 2.39

**Table 3:** Actual response in BBD for optimization of TAC-THQ-NLCs with predicted values generated by Design Expert Software.

Runs	Independent Variables			Dependent Variables							
	*X*_1_	*X*_2_	*X*_3_	*Y*_1_		*Y*_2_		*Y*_3_		*Y*_4_	
				Actual	Predicted	Actual	Predicted	Actual	Predicted	Actual	Predicted
F1	4	7	2	164.6	164.1	0.129	0.127	70.38	70.02	77.16	76.95
F2	3	6	4	90.5	91.01	0.175	0.175	58.73	58.35	66.16	66.1
F3	4	5	4	145.1	145.6	0.227	0.228	65.21	65.57	73.21	73.42
F4	5	6	4	175.2	175.1	0.301	0.297	75.66	75.56	82.13	82.27
F5	5	7	3	185.9	186.9	0.212	0.214	80.98	80.96	86.46	86.61
F6	5	6	2	202.9	202.4	0.217	0.216	82.17	82.55	87.39	87.45
F7	3	5	3	116.5	115.5	0.146	0.144	64.19	64.21	72.12	71.97
F8	4	6	3	129.8	127.7	0.151	0.154	70.98	71.06	77.02	76.75
F9	4	6	3	127.6	127.7	0.155	0.154	71.54	71.06	76.36	76.75
F10	4	7	4	137.6	136.7	0.195	0.197	63.94	64.07	71.82	71.53
F11	4	5	2	175.7	176.6	0.171	0.169	71.66	71.53	77.96	78.25
F12	3	6	2	122.1	122.2	0.123	0.127	63.16	63.26	71.32	71.18
F13	4	6	3	125.7	127.7	0.157	0.154	70.67	71.06	76.88	76.75
F14	5	5	3	204.1	203.7	0.261	0.263	83.95	83.69	89.17	88.82
F15	3	7	3	110.4	110.8	0.123	0.121	63.68	63.94	70.66	71.01

*X*_1_ = Lipid mixture (% *w*/*v*); *X*_2_ = Surfactant mixture (% *w*/*v*); *X*_3_ = Sonication time (minute); *Y*_1_ = Particle size (nm); *Y*_2_ = Polydispersity index (PDI); *Y*_3_ = % Entrapment efficiency-TAC; *Y*_4_ = % Entrapment efficiency-THQ.

**Table 4:** Summary of the regression analysis for PS, PDI, and EE responses for fitting the data to various models.

Model	R^2^	R^2^ Adjusted	R^2^ Predicted	SD	Adequate Precision	Remark
Response (*Y*_1_): PS						
Linear	0.9015	0.8746	0.8583	12.38	83.7893	-
2FI	0.9040	0.8321	0.7804	14.33		-
Quadratic	0.9992	0.9978	0.9941	1.65		Suggested
Cubic	0.995	0.9966	-	2.05		Aliased
Response (*Y*_2_): PDI						
Linear	0.8744	0.8402	0.7950	0.0209	55.5678	-
2FI	0.8862	0.8008	0.6626	0.0233		-
Quadratic	0.9980	0.9945	0.9751	0.0039		Suggested
Cubic	0.9995	0.9966	-	0.0031		Aliased
Response (*Y*_3_): EE-TAC						
Linear	0.9202	0.8984	0.8309	2.42	65.3883	-
2FI	0.9234	0.8660	0.5998	2.78		-
Quadratic	0.9986	0.9961	0.9842	0.4752		Suggested
Cubic	0.9995	0.9966	-	0.4409		Aliased
Response (*Y*_4_): EE-THQ						
Linear	0.9296	0.9105	0.8521	2.00	68.4019	-
2FI	0.9304	0.8782	0.6328	2.34		-
Quadratic	0.9987	0.9963	0.9842	0.4068		Suggested
Cubic	0.9996	0.9973	-	0.3478		Aliased

**Table 5:** Optimized levels of independent factors and their corresponding predicted and experimental outcomes.

Independent Variables	Optimized Level	Outcomes	Predicted	Experimental
Lipid-mixture (%)	4.36	PS (nm)	147.98	144.95 ± 2.80
Surfactants (%)	6.26	PDI	0.164	0.160 ± 0.021
Sonication time (min)	2.73	EE-TAC (%) EE-THQ (%)	75.03 80.35	73.44 ± 2.54 78.62 ± 2.75

**Table 6:** Results from in vitro drug-release kinetics modelling of TAC-THQ-NG.

TAC-THQ-NG	Release Model	Conc-Time Equation	R^2^	Release Exponent (n)
TAC	Zero order	D_t_ = D_0_ + k_0_t	0.7843	
	1st order	ln D_t_ = lnD_0_ + k_1_t	0.8741	
	Higuchi matrix	D_t_ = D_0_ + kt^1/2^	0.9388	
	Korsmeyer–Peppas	A_t_/D_∞_ = kt^n^	0.9892	0.2511
THQ	Zero order	D_t_ = D_0_ + k_0_t	0.8095	
	1st order	ln D_t_ = lnD_0_ + k_1_t	0.9258	
	Higuchi matrix	D_t_ = D_0_ + kt^1/2^	0.955	
	Korsmeyer–Peppas	A_t_/D_∞_ = kt^n^	0.9822	0.2409

**Table 7:** Stability of TAC-THQ-NLCs and TAC-THQ-NG in different storage conditions.

Storage Condition	Time (Months)	TAC-THQ-NLCs				TAC-THQ-NG			
		PS (nm)	PDI	%EE-TAC	%EE-THQ	Drug Content (%-TAC)	Drug Content (%-THQ)	pH	Spreadability
Initial	0	144.95 ± 2.80	0.160 ± 0.021	73.44 ± 2.54	78.62 ± 2.75	97.99 ± 1.79	98.51 ± 1.33	6.72 ± 0.29	8.10 ± 0.46
5 ± 3 °C	3	147.22 ± 2.81	0.172 ± 0.020	71.42 ± 2.49	76.63 ± 2.31	96.65 ± 1.85	97.11 ± 1.57	6.54 ± 0.32	7.07 ± 0.40
25 ± 2 °C	3	152.03 ± 3.16	0.183 ± 0.018	69.68 ± 2.70	74.15 ± 2.86	95.59 ± 1.78	95.02 ± 1.67	6.42 ± 0.28	7.83 ± 0.42

**Table 8:** Independent and dependent factors with their actual levels (coded) and desired outcomes in the BBD for optimization of TAC-THQ-NLCs.

Factor	Actual Levels (Coded)		
Independent Factors	Low (−1)	Medium (0)	High (+1)
*X*_1_ = Lipid mixture (%*w*/*v*)	3	4	5
*X*_2_ = Surfactant mixture (%*w*/*v*)	5	6	7
*X*_3_ = Sonication time (minute)	2	3	4
Dependent factors	Desired outcome		
*Y*_1_ = Particle size (nm)	Minimize		
*Y*_2_ = Polydispersity index (PDI)	Minimize		
*Y*_3_ = % Entrapment efficiency-TAC	Maximize		
*Y*_4_ = % Entrapment efficiency-THQ	Maximize		

## Data Availability

All data that supports the findings of this study are included within the article.
